# TRANSAID: a hybrid deep learning framework for translation site prediction with integrated biological feature scoring

**DOI:** 10.3389/fbinf.2025.1676149

**Published:** 2026-01-19

**Authors:** Yan Li, Boran Wang, Zhen Liu, Wei Wei, Caiyi Fei, Shi Xu, Tiyun Han, Wei Geng, Zengding Wu

**Affiliations:** 1 Department of Breast Surgery, Peking Union Medical College Hospital, Chinese Academy of Medical Sciences and Peking Union Medical College, Beijing, China; 2 Department of International Medical Service (Xidan Campus), Peking Union Medical College Hospital, Chinese Academy of Medical Sciences and Peking Union Medical College, Beijing, China; 3 Breast Disease Diagnosis and Treatment Center, Affiliated Hospital of Qinghai University, Affiliated Cancer Hospital of Qinghai University, Xining, China; 4 Beijing Tiantan Hospital, Capital Medical University, Beijing, China; 5 Beijing Friendship Hospital, Capital Medical University, Beijing, China; 6 Department of AI and Bioinformatics, Nanjing Chengshi Biopharmaceutical (TheraRNA) Co., Ltd., Nanjing, China

**Keywords:** translation site prediction, deep learning, open reading frame, integrated scoring system, cross-species analysis, transcriptome annotation

## Abstract

**Introduction:**

Translation initiation and termination are critical regulatory checkpoints in protein synthesis, yet accurate computational prediction of their sites remains challenging due to training data biases and the complexity of full-length transcripts.

**Methods:**

To address these limitations, we present TRANSAID (TRANSlation AI for Detection), a novel deep learning framework that accurately and simultaneously predicts translation initiation (TIS) and termination (TTS) sites from complete transcript sequences. TRANSAID’s hierarchical architecture efficiently processes long transcripts, capturing both local motifs and long-range dependencies. Crucially, the model was trained on a human transcriptome dataset that was rigorously partitioned at the gene level to prevent data leakage and included both protein-coding (NM) and non-coding (NR) transcripts.

**Results:**

This mixed-training strategy enables TRANSAID to achieve high fidelity, correctly identifying 73.61% of NR transcripts as non-coding. Performance is further enhanced by an integrated biological scoring system, improving “perfect ORF prediction” for coding sequences to 94.94% and “correct non-coding prediction” to 82.00%. The human-trained model demonstrates remarkable cross-species applicability, maintaining high accuracy on organisms from mammals to yeast. Beyond annotation, TRANSAID serves as a powerful discovery tool for novel coding events. When applied to long-read sequencing data, it accurately identified previously unannotated protein isoforms validated by mass spectrometry (76.28% validation rate). Furthermore, homology searches of high-scoring ORFs predicted within NR transcripts suggest a strong potential for identifying cryptic translation events.

**Discussion:**

As a fully documented open-source tool with a user-friendly web server, TRANSAID provides a powerful and accessible resource for improving transcriptome annotation and proteomic discovery.

## 1 Introduction

Translation initiation and termination represent critical regulatory checkpoints in protein synthesis, fundamentally determining both the quantity and diversity of the cellular proteome (Sonenberg and Hinnebusch, 2009; Jackson et al., 2010). The dysregulation of translation is implicated in a wide array of human pathologies, including cancer (Jiang et al., 2021; Truitt and Ruggero, 2016), neurodegenerative disorders (Skariah and Todd, 2021), and viral infections (Garcia‐Moreno et al., 2018). Consequently, the translation machinery has emerged as a promising target for therapeutic intervention, with inhibitors targeting initiation factors showing potential as anticancer agents (Andreev et al., 2012). Furthermore, the discovery of cryptic translation events, which produce immunogenic peptides from previously unannotated regions, has opened new avenues for cancer immunotherapy (Li et al., 2024). Aberrant transcripts in cancer can serve as a rich source of tumor neoantigens; however, identifying their protein products is a significant bottleneck, necessitating high-accuracy prediction algorithms to bridge the gap between transcriptomic data and proteomic validation (Ji et al., 2025).

The precise identification of translation initiation sites (TIS) and termination sites (TTS) is therefore essential for elucidating gene expression mechanisms and characterizing the full complexity of cellular proteomes (Ingolia et al., 2009). Recent advancements in long-read sequencing technologies, such as those from Pacific Biosciences and Oxford Nanopore, have significantly enhanced our ability to capture full-length transcript sequences (Wenger et al., 2019; Workman et al., 2019). While these technologies provide an unprecedented view of the transcriptome’s diversity, they also highlight a critical challenge: accurately identifying functional translation sites within novel transcripts that lack established annotations (Chen et al., 2020). The ever-expanding repository of transcript data thus demands robust and scalable computational approaches that can reliably predict translation sites *de novo*.

The regulation of translation in eukaryotes presents multiple layers of complexity. While the classical scanning model proposes that initiation typically occurs at the first AUG codon encountered by the ribosome (Kozak, 1986), mounting evidence reveals widespread use of alternative initiation codons and the functional importance of upstream open reading frames (uORFs), which significantly modulate the expression of primary ORFs (Starck et al., 2016; Spealman et al., 2018). Translation initiation is a highly context-dependent process, profoundly influenced by sequence motifs like the Kozak consensus sequence and intricate RNA secondary structures within the 5′ untranslated region (Hinnebusch et al., 2016; Kozak, 2005). Further complicating this landscape is the recent discovery of functional micropeptides translated from transcripts previously classified as non-coding RNAs (ncRNAs), challenging the conventional binary distinction between coding and non-coding genes (van Heesch et al., 2019). This biological complexity is compounded by epitranscriptomic modifications, such as N6-methyladenosine (m6A), which create a dynamic regulatory network that fine-tunes translation efficiency in response to cellular cues (Tian et al., 2021).

Computational approaches to translation prediction have evolved substantially to address these challenges. Early methods relied on statistical models and sequence-based features, such as the position weight matrices used by NetStart (Pedersen and Nielsen, 1997) and ATGpr (Salamov et al., 1998). Subsequent machine learning approaches, including support vector machines used in tools like StartCodon (Liu and Wong, 2003) and TISRover (Saeys et al., 2007), integrated a broader range of features but often faced scalability limitations with large transcriptomic datasets.

More recently, deep learning has emerged as a powerful paradigm for this task (Wang et al., 2025). Tools such as TITER employ sophisticated architectures, like a combination of convolutional and recurrent neural networks (CNN-BiLSTM), to achieve high precision in scoring candidate TIS locations (Zhang et al., 2017). However, as a specialized TIS predictor, TITER does not identify the corresponding TTS, and thus cannot predict the full ORF or its protein product. On the other end of the spectrum, statistical model-based tools like GeneMarkS-T utilize Hidden semi-Markov Models (HSMMs) with an unsupervised self-training strategy to parse full transcripts into coding and non-coding regions (Tang et al., 2015). While robust, these models may not capture the complex, non-linear sequence patterns that deep learning architectures excel at. The more recent deep learning framework, TranslationAI, utilizes a CNN to predict TIS-TTS pairs from full-length transcripts (Fan et al., 2025). However, a critical limitation of many existing methods, including TranslationAI, is their insufficient training on non-coding (NR) transcripts. This biases the models toward overpredicting translation events, resulting in a high rate of false positives when analyzing the vast non-coding transcriptome.

Despite these advances, current approaches often exhibit one or more significant limitations: a persistent bias towards protein-coding sequences, the independent prediction of TIS and TTS without enforcing biological constraints, substantial computational demands, struggles with processing full-length transcripts without truncation, and inadequate integration of known biological features.

To address these limitations, we present TRANSAID, a comprehensive deep learning framework for the simultaneous prediction of TIS and TTS pairs from full-length eukaryotic transcripts. TRANSAID employs a hierarchical architecture combining embedding layers with dilated convolutions and residual connections, enabling efficient and accurate processing of complete transcripts while capturing both local motifs and long-range dependencies. Crucially, by training on a balanced dataset of both coding (NM) and non-coding (NR) transcripts and implementing a novel biologically-informed scoring system, TRANSAID significantly reduces false positive predictions and improves overall accuracy. In this study, we demonstrate TRANSAID’s superior performance, its ability to generalize across species, its capacity to learn fundamental biological rules, and its practical application in novel protein discovery.

## 2 Materials and methods

### 2.1 Dataset preparation and splitting

Data Source and Initial Processing: All transcript sequences and annotations used in this study were sourced from the UCSC Genome Browser database. We downloaded the human reference transcripts FASTA file (GRCh38_latest_rna.fna) and the corresponding comprehensive gene annotation file (GRCh38_latest_rna.gbff), which are based on the GRCh38/hg38 assembly. This curated dataset utilizes RefSeq identifiers (e.g., NM_, NR_) while employing the chr chromosome naming convention.

From the annotation file, we parsed and extracted essential information for each transcript, including its unique identifier (without the version suffix), the corresponding gene symbol, and, for protein-coding transcripts, the start and end coordinates of its primary coding sequence (CDS).

Transcript Classification and Dataset Splitting: Transcripts were classified into two primary categories based on their RefSeq identifier prefix: protein-coding (transcripts with NM_ prefixes) and non-coding (transcripts with NR_ prefixes). It is noteworthy that some transcripts annotated as non-coding may contain functional small open reading frames (sORFs). While we did not exclude these potential sORF-containing transcripts from the non-coding set during training, we address this biological complexity through a dedicated downstream analysis presented in the Results section (Supplementary Table S1).

To ensure a rigorous and unbiased evaluation of our model’s generalization capability, the entire dataset was partitioned at the gene level. All transcript isoforms belonging to a single gene were exclusively assigned to only one of the data splits. This strict partitioning prevents data leakage between the training and evaluation sets, a critical step for validating model performance on unseen genes rather than merely on unseen isoforms. The dataset was split into a training set (80% of genes) and a held-out validation/test set (20% of genes). This 80:20 split was chosen to maximize the amount of data available for model training while retaining a substantial, independent set for robust performance assessment. All final performance metrics and comparisons reported in this study were evaluated on this 20% held-out set.

Data Encoding and Representation: For model input, RNA sequences were transformed into numerical format using integer encoding, where A, C, G, T/U were mapped to 1, 2, 3, and 4, respectively. Ambiguous nucleotides (N) and padding were mapped to 0. When combined with an embedding layer, this integer encoding, is generally considered to offer computational and memory efficiency benefits over traditional one-hot encoding, especially for long sequences. This approach transforms sparse, high-dimensional representations into dense, lower-dimensional vectors that are often more computationally tractable and capable of capturing relevant sequence patterns (Mikolov et al., 2013; Asgari and Mofrad, 2015; Yue and Wang, 2018).

Output labels were represented as three-dimensional one-hot vectors for each nucleotide position, corresponding to three mutually exclusive classes: translation initiation site (TIS) as [1,0,0], translation termination site (TTS) as [0,1,0], and non-special positions as [0,0,1]. This encoding scheme ensures that there is no ordinal relationship between the classes. To accommodate the varying lengths of transcripts, a maximum sequence length was determined based on the 99.9th percentile of the human transcriptome length distribution (27,112 nt). Shorter sequences were padded with the 0 value to this length, while longer sequences were truncated.

### 2.2 TRANSAID model architecture

**FIGURE 1:**
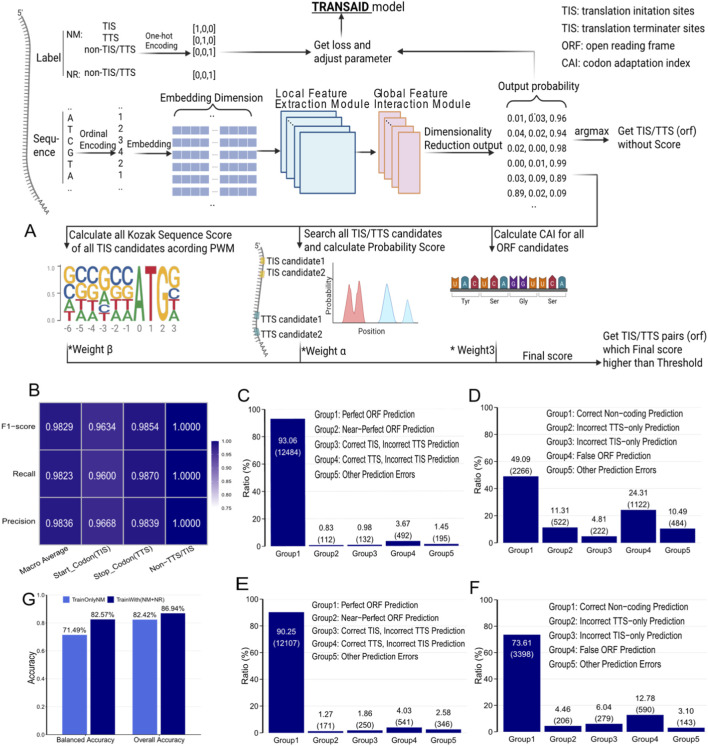
TRANSAID Architecture and Performance on Human Transcripts. **(A)** Schematic of the TRANSAID framework. The model processes RNA sequences through an embedding layer and a hybrid convolutional neural network (CNN) with residual blocks. The output probabilities are integrated with biological scores (Kozak context, CAI) through a weighted system to predict final ORFs. **(B)** Position-level performance of the TrainNMonly model, showing high F1-scores for TIS and TTS identification. **(C,D)** ORF-level performance of the TrainNMonly model on protein-coding (NM) and non-coding (NR) transcripts, respectively. The model achieves 93.06% “Perfect ORF” prediction on NM transcripts but correctly identifies only 49.09% of NR transcripts as non-coding. **(E,F)** Performance of the Train (NM + NR) model, which shows a slight decrease in “Perfect ORF” prediction (90.25%) but a substantial improvement in “Correct Non-coding” classification (73.61%). **(G)** Comparison of training strategies, demonstrating the superior overall and balanced accuracy of the mixed Train (NM + NR) model.

The TRANSAID model implements a hierarchical deep learning architecture that integrates a neural network for sequence analysis with a downstream biological feature scoring system (Figure 1A). The deep learning component is composed of four main modules, designed to efficiently process full-length transcripts and capture both local and global sequence features. The architecture is based on the TRANSAID_Embedding model from our training scripts.

1. Sequence Embedding Layer: This initial module maps the discrete integer-encoded input sequence into a continuous, high-dimensional vector space. It consists of an embedding layer that transforms each nucleotide integer (0–4) into a 128-dimensional vector representation. The padding_idx = 0 parameter ensures that all padded positions have a zero vector representation. Preventing them from contributing to the gradient during training. The 128-dimensional embedding vectors for each nucleotide (A, C, G, T/U) are initially randomly generated. These vectors are then iteratively learned and optimized through backpropagation during the model training process, allowing them to capture nuanced biochemical and structural properties relevant to translation initiation and termination.
2. Local Feature Extraction Module: This initial module is designed to extract fundamental local sequence features from the high-dimensional nucleotide embeddings. It consists of a single 1D convolutional layer (self.conv1) with an input channel dimension of 128 (from the embedding layer) and an output of 32 filters, using a kernel width of 3 and padding = “same”. The output of this convolutional layer is then subjected to Batch Normalization (self.bn1) and a ReLU activation function (self.relu). This sequential process transforms the 128-dimensional embedding into a 32-dimensional feature map, capturing basic, short-range local sequence patterns (e.g., specific nucleotide triplets or signals) and introducing non-linearity.
3. Global Feature Interaction Module: To capture long-range dependencies across the entire transcript, this module employs a deep stack of residual blocks with dilated convolutions (Supplementary Figure S1). The architecture consists of three sequential stages, each comprising four ResidualBlock units.

Stage 1: This stage takes the 32-dimensional feature map from the Local Feature Extraction Module. It comprises four ResidualBlock units, each utilizing 1D convolutions with a kernel width of 26 and a dilation rate of 1.

Dimension Expansion 1: Following Stage 1, a 1x1 convolutional layer (self.conv2) is applied, which increases the feature map dimensionality from 32 to 64. This operation is immediately succeeded by Batch Normalization (self.bn2) and a ReLU activation.

Stage 2: The 64-dimensional feature map from the previous expansion then feeds into this stage. It also consists of four ResidualBlock units, but here, the 1D convolutions use a kernel width of 26 and a progressively increased dilation rate of 2.

Dimension Expansion 2: After Stage 2, another 1x1 convolutional layer (self.conv3) is used to further increase the feature map dimensionality from 64 to 128. This is also followed by Batch Normalization (self.bn3) and a ReLU activation.

Stage 3: The highest-dimensional feature map (128-dimensional) enters this final stage. It contains four ResidualBlock units, where the 1D convolutions use an even larger kernel width of 36 and the highest dilation rate of 5.

Within each ResidualBlock unit (detailed in Supplementary Figure S1), residual connections facilitate effective gradient flow during training. The progressively increasing dilation rates (1, 2, 5) within the stages, combined with large kernel widths (26 and 36), exponentially expand the receptive field without increasing computational cost, enabling the model to learn complex relationships between distant TIS and TTS signals across the entire RNA transcript. The intermittent 1x1 convolutions serve to increase the feature channel depth between stages (32 → 64 → 128), enhancing the model’s capacity to represent richer, more abstract long-range features.

4. Output Decoding Module: The final module maps the high-level 128-dimensional feature representations back to the three-class prediction space. It consists of two 1x1 convolutional layers that reduce the feature dimensionality (128 → 32 → 3), with a ReLU activation in between. The final output is a tensor of shape (batch_size, seq_len, 3), representing the logits for the TIS, TTS, and non-special classes for each nucleotide position.

### 2.3 Model training

Model training was conducted on an NVIDIA H100 GPU. We trained two main models for our analyses: a TrainNMonly model trained exclusively on protein-coding (NM) transcripts, and the final TRANSAID model trained on a mixed dataset of both NM and non-coding (NR) transcripts. All hyperparameters were kept consistent between the two training runs.

The model was trained for a maximum of 50 epochs with a batch size of 4. We used the Adam optimizer with an initial learning rate of 0.001. A learning rate decay scheduler was implemented to reduce the learning rate by a factor of 0.5 if the validation loss did not improve for 3 consecutive epochs. To prevent overfitting, an early stopping mechanism was employed, terminating the training process if the validation loss on the 20% held-out set did not improve for 5 consecutive epochs. The loss function was a standard cross-entropy loss applied to the three output classes. The model state from the epoch with the lowest validation loss was saved as the final best model for all subsequent evaluations.

### 2.4 Integrated scoring system

While the deep learning model provides nucleotide-level probabilities, translating these into the single, most biologically significant Open Reading Frame (ORF) per transcript is a non-trivial challenge that requires an additional layer of logic. To address this, we developed an integrated scoring system that first identifies all potential ORFs satisfying hard biological constraints (e.g., triplet codon structure) and then scores these candidates by combining model-derived probabilities with established biological heuristics. This system serves as a probabilistic ranking function to select the most plausible ORF. For each potential ORF, an Integrated_Score is calculated from five components, each normalized to a 0–1 scale.

#### 2.4.1 Feature components

1. TIS Probability (TIS_prob_): The raw probability for the start codon as predicted by the TRANSAID deep learning model.
2. TTS Probability (TTS_prob_): The raw probability for the stop codon as predicted by the TRANSAID deep learning model.
3. Kozak Sequence Score (Kozak__score_): The Kozak sequence is evaluated across specified positions relative to the start codon using a position weight matrix (PWM). Each position’s score is calculated as the probability of the base being present at that position, derived from human genomic sequences with effective translation initiation sites.

$${Kozak}_{score}=\Pi P\left(\left.base_{i}\right|position_{i}\right)\times 10000$$

kozak_pwm = {

−6: {‘A’: 0.22, ‘C’: 0.28, ‘G’: 0.32, ‘T’: 0.18},

−5: {‘A’: 0.20, ‘C’: 0.30, ‘G’: 0.30, ‘T’: 0.20},

−4: {‘A’: 0.18, ‘C’: 0.32, ‘G’: 0.30, ‘T’: 0.20},

−3: {‘A’: 0.25, ‘C’: 0.15, ‘G’: 0.45, ‘T’: 0.15},

−2: {‘A’: 0.20, ‘C’: 0.35, ‘G’: 0.25, ‘T’: 0.20},

−1: {‘A’: 0.20, ‘C’: 0.35, ‘G’: 0.25, ‘T’: 0.20},

0: {‘A’: 1.00, ‘C’: 0.00, ‘G’: 0.00, ‘T’: 0.00}, # A of ATG

1: {‘A’: 0.00, ‘C’: 0.00, ‘G’: 0.00, ‘T’: 1.00}, #T of ATG

2: {‘A’: 0.00, ‘C’: 0.00, ‘G’: 1.00, ‘T’: 0.00}, #G of ATG

3: {‘A’: 0.20, ‘C’: 0.20, ‘G’: 0.40, ‘T’: 0.20}

}

Here, signifies the product over each base, and $P\left(\left.base_{i}\right|position_{i}\right)$ represents the probability of base at position. For example, $P\left(\left.\prime \prime A^{\prime \prime }\right|-6\right)$ = 0.22 indicates a 0.22 probability of an ‘A’ being at position −6.

4. Codon Adaptation Index (CAI_score_): CAI provides a measure of codon usage bias relative to a set of highly expressed genes and is calculated via:

$${CAI}_{score}={\left(\prod_{i=1}^{L}w_{i}\right)}^{\frac{1}{L}}$$

Here, *L* represents the total number of codons in the identified Open Reading Frame (ORF), and *w*_*i*_ is the relative adaptiveness value for the i-th codon. Steps include ORF identification, division into codons, looking up relative adaptiveness values for each codon, calculating geometric means, and normalizing the score.

5. GC Content Score (GC_score_): GC content is evaluated by determining the proportion of G and C nucleotides within a potential ORF sequence:

$${GC}_{score}=2\times e^{{-0.5\times \left(\left(GC_{content}-0.42\right)/0.2\right)}^{2}}-1$$

This scoring uses a Gaussian model to convert GC content rates into normalized scores.

#### 2.4.2 Integrated score calculation

The Integrated Score for each ORF is calculated by combining feature scores with predefined weights, following:

$$Integrated_{score}=w_{TIS}\times TIS_{prob}+w_{TTS}\times TTS_{prob}+w_{Kozak}\times Kozak_{score}+w_{CAI}\times CAI_{score}+w_{GC}\times GC_{score}$$

#### 2.4.3 Data-driven weight optimization

To ensure the robustness and objectivity of the scoring system, the weights (w) and the final decision threshold were not set arbitrarily but were optimized through a data-driven approach. We performed an extensive grid search on the independent validation set (20% of genes) to identify the parameter combination that maximized the standard accuracy. This process systematically evaluated over 160,000 unique parameter sets. The results of this optimization confirmed that the model’s TIS/TTS probabilities are the most influential features and validated the contribution of the biological heuristics. A sensitivity analysis demonstrated that the system’s performance is stable across a plateau of near-optimal parameter values, underscoring its robustness (Supplementary Figure S2). The final, optimized parameters ( $w_{TIS}$ = 0.30, $w_{TTS}$ = 0.50, $w_{Kozak}$ = 0.04, $w_{CAI}$ = 0.04, $w_{GC}$ = 0.00, and a threshold of 0.635) were used for all subsequent analyses presented in this study.

The final Integrated Score for each ORF is calculated by combining the four impactful feature scores with their data-driven weights, following the refined formula:

$$Integrated_{score}=0.3\times TIS_{prob}+0.5\times TTS_{prob}+0.04\times Kozak_{score}+0.04\times CAI_{score}$$

### 2.5 Performance evaluation and benchmarking

The performance of all models was evaluated on the independent 20% test set of human genes. We defined a comprehensive set of metrics at the ORF level for both coding and non-coding transcripts, including Perfect ORF (defined as the precise rightly identification of both the 3-nucleotide TIS and TTS codons), Correct TIS incorrect TTS, Correct TTS incorrect TIS, Other Errors, Correct Non-coding, and False ORF.

To contextualize TRANSAID’s performance, we benchmarked it against three state-of-the-art tools: TranslationAI, GeneMarkS-T, and TITER. To ensure a fair and direct comparison, all tools were run with their default parameters on the same evaluation datasets. For the six non-human species, the complete set of NM and NR transcripts was used for evaluation. For the human benchmark, a special test set was created for each tool by excluding any transcripts that were part of its original training dataset, thus guaranteeing that each model was evaluated on data it had not seen before. Performance was primarily evaluated using the “Perfect ORF” (defined as a prediction where the final ORF, after adjustment by the integrated scoring system, correctly matches the annotated TIS and TTS.) and “Correct Non-coding” metrics.

### 2.6 Cross-species and experimental validation

To evaluate the cross-species applicability of the human-trained TRANSAID model, we collected comprehensive transcriptomic data from six additional model organisms spanning a wide evolutionary range. All data were downloaded from the NCBI Reference Sequence (RefSeq) database. For each species, we obtained the full set of curated transcript sequences (rna.fna.gz) and their corresponding annotations (rna.gbff.gz). The species and specific assemblies included in our analysis were:

1. Mus musculus (Mouse) - GRCm39 assembly
2. Danio rerio (Zebrafish) - GRCz11 assembly
3. Drosophila melanogaster (Fruit Fly) - Release 6 plus ISO1 MT assembly
4. Caenorhabditis elegans (Nematode) - WBcel235 assembly
5. Arabidopsis thaliana (Thale Cress) - TAIR10.1 assembly
6. Saccharomyces cerevisiae (Yeast) - R64 assembly

For each species, transcripts were classified as protein-coding (NM) or non-coding (NR) based on their RefSeq annotations and processed through the TRANSAID prediction pipeline.

### 2.7 Experimental validation with proteomics data

To validate TRANSAID’s utility in novel protein discovery, we analyzed publicly available PacBio Iso-Seq and mass spectrometry (MS) data from Jurkat T-cells (Miller et al., 2022). Full-length transcript sequences were processed by TRANSAID using an Integrated_Score cutoff of 0.50 to predict a non-redundant set of 17,046 protein sequences. These predicted proteins were then used as a custom reference database. We subsequently mapped the experimentally identified MS peptides from the original study against this database to determine the validation rate, assessing the percentage of our predicted proteins supported by direct peptide evidence.

### 2.8 Code and data availability

The TRANSAID software, including source code, trained models, and usage documentation, is freely available on GitHub at https://github.com/wuzengding/TRANSAID. For complete reproducibility of our study, the TRANSAID_training_latest branch contains all scripts used for model training, performance evaluation, data analysis, and figure generation. A user-friendly web server for online prediction is accessible at http://58.242.248.157:6005/.

## 3 Results

### 3.1 TRANSAID achieves high accuracy on human transcripts through mixed training and integrated scoring

#### 3.1.1 Training strategy and performance on human transcripts

The development of TRANSAID began with a meticulously prepared human transcriptome dataset sourced from the UCSC Genome Browser, ensuring a robust foundation for model training and evaluation. This dataset comprised both manually curated protein-coding (NM) and non-coding (NR) transcripts, meticulously partitioned at the gene level into distinct training (80%) and held-out validation/test (20%) sets to prevent data leakage and enable unbiased performance assessment. Prior to training, transcript sequences were integer-encoded and labeled with three-dimensional one-hot vectors for TIS, TTS, and non-special positions, accommodating transcript length variations up to 27,112 nucleotides (99.9th percentile) through padding or truncation (Supplementary Figure S3A).

Initial model training focused exclusively on protein-coding transcripts (NM class), resulting in the TrainNMonly version of TRANSAID. This model demonstrated strong classification capabilities at the nucleotide level. A confusion matrix analysis of its performance on 13,415 human NM test transcripts revealed exceptionally high F1-scores of 96.34% for TIS, 98.54% for TTS, and nearly 99.99% for non-translation sites (Figure 1B). Converting these nucleotide-level metrics to an Open Reading Frame (ORF) level assessment provided a more holistic view of translation prediction accuracy. The TrainNMonly model achieved a “Perfect ORF Prediction” (defined as the precise right identification of both the 3-nucleotide TIS and TTS codons) rate of 93.06% for NM transcripts, indicating complete agreement between predicted and actual TIS/TTS positions across the entire transcript. Furthermore, 0.83% of transcripts exhibited “Near-Perfect ORF Prediction” (minimal errors with single nucleotide deviations), while 0.98% had correctly predicted TIS only, and 3.67% had correctly predicted TTS only (Figure 1C).

However, a critical limitation emerged when this TrainNMonly model was applied to predict ORFs in human non-coding (NR) transcripts. We observed a significantly elevated false positive rate at the nucleotide level, where numerous non-special sites were misclassified as TIS or TTS, resulting in poor prediction metrics (Supplementary Figures S3c,d). This substantial overprediction suggested a fundamental bias inherent in training exclusively on protein-coding data. This underlying misclassification at the nucleotide level led to a significantly elevated false positive rate at the ORF level, with only 49.09% of NR transcripts correctly identified as non-coding (Figure 1D). Alarmingly, 24.31% of NR transcripts were erroneously predicted to contain complete ORFs (False ORF), while 11.31% were incorrectly predicted to contain only TTS, and 4.81% only TIS. This substantial overprediction of translation events in non-coding sequences suggested a fundamental bias inherent in training exclusively on protein-coding data. Such a model, lacking exposure to the diverse characteristics of non-coding transcripts during training, struggles to accurately distinguish genuine translation signals from spurious motifs in non-coding contexts, leading to artificially inflated translation predictions.

To address this limitation and improve the model’s ability to differentiate between truly coding and non-coding sequences, we subsequently trained the Train (NM + NR) model. This version integrated 18,462 NR transcripts into the training dataset alongside NM transcripts, ensuring both types were proportionally distributed across each training batch. Re-evaluation of the Train (NM + NR) model’s performance confirmed the effectiveness of this mixed training strategy. While the “Perfect ORF Prediction” rate for NM test data marginally decreased from 93.06% to 90.25% (Figure 1E), this slight reduction in coding accuracy was offset by a substantial improvement in non-coding classification. The proportion of NR transcripts correctly identified as non-coding dramatically increased from 49.09% to 73.61% (Figure 1F). When combining both NM and NR test data, the model’s overall accuracy improved from 82.42% to 86.94%. Furthermore, to account for the inherent class imbalance in the test set (where NM transcripts are more numerous), we constructed a balanced test set with equal numbers of NM and NR samples. On this balanced set, the Train (NM + NR) model’s Balanced Accuracy improved by 11.08%, from 71.49% to 82.57% (Figure 1G), unequivocally demonstrating the critical importance of incorporating both coding and non-coding transcripts during the training phase to develop a robust and accurate translation site prediction model.

#### 3.1.2 Optimizing ORF selection with an integrated scoring system

Despite the significant overall performance enhancement achieved by the Train (NM + NR) model, approximately 10% of transcripts still presented incorrect classifications. To further refine our predictions and address these residual errors, we developed an integrated scoring system. This system leverages an additional layer of biological logic to enhance accuracy, moving beyond a simple maximum-probability selection.

##### 3.1.2.1 Analysis of prediction errors

A detailed analysis revealed that the nature and severity of these prediction errors varied. For instance, in “Near-Perfect ORF Predictions” for protein-coding (NM) transcripts, only 1-2 out of 6 nucleotide positions might be incorrect, while the remaining 4-5 positions were correctly identified. Such minor deviations, often correctable using prior biological knowledge of codon structure, differ fundamentally from Correct TIS, incorrect TTS Prediction or Correct TTS, incorrect TIS Prediction errors. Similarly, Incorrect TIS-only Prediction errors in non-coding (NR) transcripts might involve only 2-3 nucleotides incorrectly predicted as TIS without corresponding TTS sites – errors that could potentially be filtered using the biological constraint that functional ORFs require a termination codon. To develop comprehensive validation and filtering rules, we performed a detailed error pattern analysis at the nucleotide level.

**FIGURE 2:**
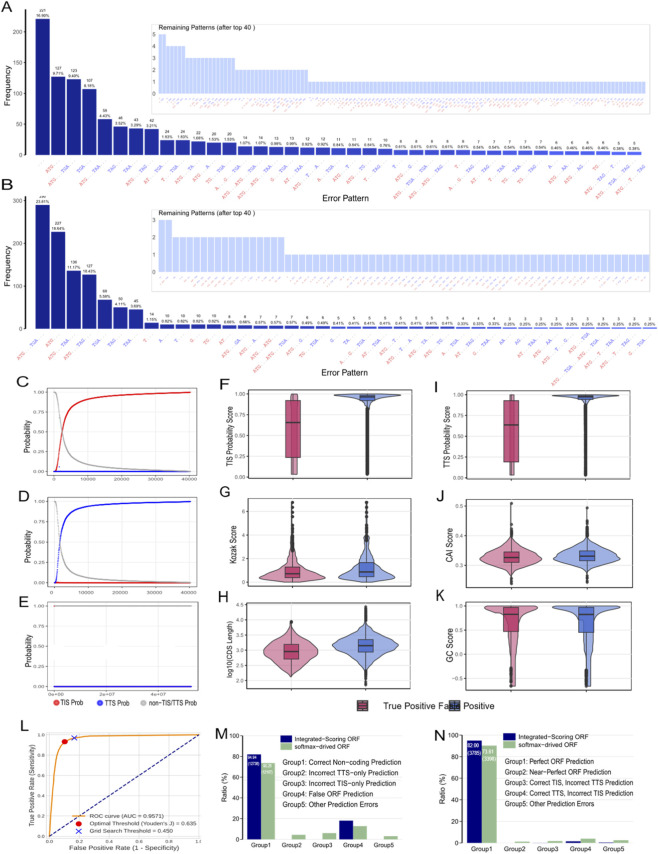
Error Analysis and Performance Optimization with Integrated Scoring. **(A,B)** Frequency distribution of error patterns in incorrectly predicted NM and NR transcripts. **(C–E)** Probability distributions for TIS, TTS, and non-TIS/TTS states across all nucleotide positions with a signal probability >0.001. The overlapping distributions highlight the limitations of a maximum-value selection approach. **(F–K)** Comparative distributions of key biological metrics between true positive ORFs (in NM transcripts) and false positive ORFs (predicted in NR transcripts), providing the basis for the integrated scoring system. **(L)** ROC curve analysis of the final Integrated_Score on the validation set, demonstrating its strong discriminatory power (AUC = 0.957). The optimal threshold determined by maximizing Youden’s J is marked. **(M,N)** The integrated scoring system with an optimized threshold (0.52) significantly boosts “Perfect ORF Prediction” on NM transcripts from 90.25% to 94.94% and “Correct Non-coding Prediction” on NR transcripts from 73.61% to 82.00%.

We extracted and quantified the most frequent error patterns observed in both incorrectly predicted NM and NR transcripts (Figures 2A,B). For NM transcripts, the top 10 error patterns included instances where no positions were predicted as TIS/TTS (−), or partial predictions like ATG- (TIS predicted, no TTS). The most frequent pattern (−), accounting for 16.90% of all errors, represented transcripts where no positions were predicted as TIS/TTS. Upon examining the probability distributions at each position for these transcripts, we frequently found significantly elevated probabilities at true TIS/TTS sites compared to surrounding regions (Supplementary Figure S4A,B). For example, in transcript NM_001003684 (Figure 2A; Table 1), positions 30–32 showed ProbTIS values of 0.03–0.06, and positions 216–218 showed ProbTTS values of 0.13–0.9, both significantly higher than surrounding regions.

**TABLE 1:** Probability matrix for NM_001003684 at key positions.

$Pos\;\left[{Prab}_{TIS},{Prab}_{TIS},{\;Prab}_{non-TIS/TTS}\;\right]$
$30\;\left[3.314e-02,1.099e-04,9.667e-01\right]$
$31\;\left[6.293e-02,1.729e-04,9.368e-01\right]$
$32\;\left[4.755e-02,1.027e-04,9.523e-01\right]$
$216\;\left[1.005e-05,1.334e-01,8.666e-01\right]$
$217\;\left[1.341e-04,9.223e-02,9.076e-01\right]$
$218\;\left[3.026e-05,1.171e-01,8.827e-01\right]$

However, these positions were ultimately classified as non-TIS/TTS due to the softmax operation’s tendency to inflate Probnon-TIS/TTS values, often exacerbated by class imbalance in the training data. Case-by-case examination (detailed in Supplementary File A) revealed that such false negatives in NM transcripts primarily resulted from the softmax operation’s stringent maximum-value selection criterion.

For NR transcripts, the top error pattern was ATG-TGA-, representing 23.81% of errors, indicating frequent erroneous prediction of a complete ORF. Detailed examination (as shown in Supplementary Figure S4C–F, and further summarized in Supplementary File B) revealed that many apparent TIS-TTS pairs in non-coding transcripts did not form valid triplet codons, suggesting that a simple codon structure verification could effectively filter numerous false positives.

##### 3.1.2.2 Developing the integrated scoring system

These observations highlighted the need for a more sophisticated post-prediction filtering approach, beyond a simple maximum-probability selection. To refine ORF prediction and enhance overall accuracy, we developed an integrated scoring system leveraging multiple biologically-informed metrics. This system assigns a quantitative “Integrated_Score” to each candidate ORF, allowing for straightforward ranking and selection of the most plausible translation events based on a combined assessment of deep learning predictions and established biological heuristics. To visualize the probability distributions, we plotted the three probability values for positions with ProbTIS >0.001 and ProbTTS >0.001 (Figures 2C–E). Both distributions exhibited an initial plateau for probabilities <0.01, followed by rapid growth between 0.01–0.75, and another plateau above 0.75. The presence of a “gray zone” in the 0.01–0.75 range indicated the need for more sophisticated thresholding approaches.

We incorporated additional biologically-informed metrics into our prediction framework. The Kozak sequence context is crucial for authentic TIS identification (Kozak, 2005), with specific scoring methods established (Grzegorski et al., 2014). The Codon Adaptation Index (CAI) reflects codon usage bias related to translation efficiency (Sharp and Li, 1987), with genuine protein-coding sequences exhibiting species-specific CAI distributions. Additionally, GC content and CDS length provide discriminative power, as functional coding sequences generally maintain species-appropriate GC content and exceed minimum length thresholds (Pozzoli et al., 2008; Swinburne et al., 2006). We compared the distributions of these metrics between true positive ORFs (in NM transcripts) and false positive ORFs (predicted in NR transcripts) (Figures 2F–K), observing significant differences, especially in TIS/TTS probabilities, Kozak score, CAI, and GC content, which provided the empirical basis for our integrated scoring system.

##### 3.1.2.3 Application and performance improvement

The Integrated_Score is calculated as a weighted sum of the model’s predicted TIS and TTS probabilities, along with Kozak score, CAI score, and GC content score. Each component is normalized to a 0–1 scale before integration (Section 2.4). We constructed ROC curves to determine the optimal threshold for filtering ORF candidates, which validated against annotated ORFs across both NM and NR transcripts (Figure 2L). Analysis revealed an optimal cutoff value of 0.5. Applying this recommended cutoff significantly improved performance compared to the raw softmax-derived approach. For NM transcripts, “Perfect ORF Prediction” increased from 90.25% to 94.94%, while for NR transcripts, “Correct Non-coding Prediction” improved from 73.61% to 82.00% (Figures 2M,N). These results unequivocally demonstrate that our integrated biological feature scoring system effectively leverages both model predictions and biological knowledge to enhance ORF identification accuracy, particularly in reducing false positives among non-coding sequences.

### 3.2 Perturbation experiments reveal TRANSAID has learned fundamental principles of translation

To ascertain whether TRANSAID’s predictive power stems from a deep understanding of biological principles or merely from learning superficial sequence patterns, we conducted a series of systematic perturbation experiments. By introducing targeted modifications into different regions of both protein-coding (NM) and non-coding (NR) transcripts from the human test set, we could assess the model’s sensitivity to changes that either respect or violate fundamental rules of translation. To isolate the direct effects of sequence alterations on the neural network’s learning, all evaluations in this section were performed using the raw model output, prior to the application of the integrated scoring system. These experiments provide compelling insights into the features the model has learned to recognize as critical for defining a functional open reading frame

#### 3.2.1 Effects of UTR mutations on protein-coding transcript prediction

**FIGURE 3:**
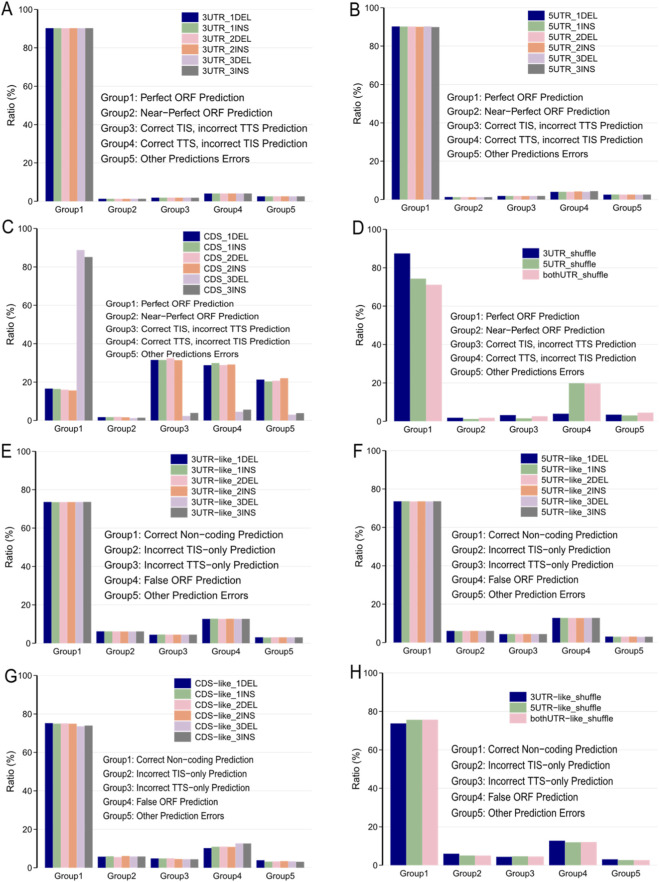
Exploring TRANSAID’s Learned Translation Features via Sequence Perturbation. **(A,B)** Performance on NM transcripts after introducing 1-3 bp insertions and deletions into 3′UTR and 5′UTR regions. Modifications to the 3′UTR had a negligible effect, while 5′UTR alterations caused a slight reduction in accuracy. **(C)** Performance after CDS region modifications. Single and double base-pair alterations causing frameshifts dramatically reduced accuracy to ∼16%, whereas in-frame 3 bp alterations maintained high performance (∼85%). **(D)** Shuffling the 5′UTR caused a more significant performance drop (74.31% “Perfect ORF”) compared to shuffling the 3′UTR (87.45%), underscoring the importance of 5′UTR sequence motifs. **(E–H)** Performance on NR transcripts remained stable across most perturbation experiments, demonstrating the model’s robustness in correctly identifying non-coding sequences.

We first investigated the model’s response to alterations within the untranslated regions (UTRs) of NM transcripts. We introduced random 1, 2, and 3 base-pair insertions and deletions at arbitrary positions within the 5′UTR and 3′UTR. The results revealed a distinct differential sensitivity. Modifications within the 3′UTR had a negligible impact on the model’s performance; the “Perfect ORF Prediction” rate remained exceptionally stable at approximately 90.24%, nearly identical to the baseline performance on unmodified sequences (Figure 3A, compare with Figure 1E). This robustness suggests that the model correctly learned that the precise nucleotide sequence of the 3′UTR, barring major structural changes, has minimal direct influence on the definition of the primary ORF’s boundaries.

In stark contrast, the model exhibited higher sensitivity to changes in the 5′UTR. While 1 bp alterations had a modest effect, 2 and 3 bp insertions led to a discernible, albeit slight, reduction in “Perfect ORF Prediction” accuracy to 89.93% (Figure 3B). This heightened sensitivity aligns perfectly with established biological knowledge: the 5′UTR is a critical regulatory region containing essential elements for translation initiation, including the Kozak consensus sequence and binding sites for initiation factors, which guide the ribosome to the correct start codon. The model’s response indicates that it has learned to recognize the importance of sequence integrity in this region.

To further probe the significance of nucleotide arrangement versus mere composition, we performed more substantial perturbation experiments involving complete shuffling of the UTR sequences while preserving their GC content. Shuffling the entire 3′UTR sequence resulted in a moderate decrease in prediction accuracy to 87.45%. However, shuffling the 5′UTR sequence caused a dramatic drop in “Perfect ORF Prediction” to just 74.31% (Figure 3D). This nearly 16-percentage-point decrease upon 5′UTR shuffling provides unequivocal evidence that TRANSAID has learned to recognize specific, position-dependent sequence motifs and structural contexts within the 5′UTR that are indispensable for proper TIS identification, a finding consistent with the known roles of elements like uORFs and IRESs (Hinnebusch et al., 2016).

#### 3.2.2 Extreme sensitivity to frameshift mutations in the CDS

Next, we assessed the model’s sensitivity to mutations within the coding sequence (CDS) itself. The results of this experiment were striking and provided the strongest evidence that TRANSAID has internalized the triplet nature of the genetic code. When 1 or 2 base-pair insertions or deletions were introduced—mutations that cause a frameshift—the model’s performance was severely compromised. “Perfect ORF Prediction” rates plummeted to a mere 15.67%–16.45% from the original 90.25% (Figure 3C). This extreme sensitivity demonstrates that the model recognizes that frameshift mutations lead to a catastrophic alteration of the downstream amino acid sequence, typically resulting in premature stop codons and non-functional protein products.

Conversely, when in-frame 3 bp insertions or deletions were introduced, the model displayed remarkable robustness. Performance remained high, with “Perfect ORF Prediction” rates of 85.14% and 88.78%, respectively (Figure 3C). This tolerance to in-frame mutations shows that the model understands that such changes correspond to the insertion or deletion of a single amino acid, a modification that often preserves the integrity of the overall protein structure and function. This differential response to in-frame versus out-of-frame mutations strongly mirrors their biological consequences (Singh and Jain, 2015) and serves as powerful validation that TRANSAID’s learning extends beyond simple pattern matching to encompass the fundamental logic of the genetic code.

#### 3.2.3 Robustness of non-coding transcript classification

Finally, we applied similar perturbations to non-coding (NR) transcripts to test whether random sequence alterations could induce erroneous ORF predictions. For these transcripts, which lack defined UTR and CDS regions, we designated the terminal 5% of the sequence as “5′UTR-like,” the middle 90% as “CDS-like,” and the terminal 5% as “3′UTR-like.” Across nearly all experiments, including small insertions/deletions and complete shuffling of the UTR-like regions, the model’s performance was virtually unaffected. The “Correct Non-coding Prediction” rate remained stable at approximately 73.60%, matching the baseline performance (Figures 3E–H, compare with Figure 1F). Only minor reductions in accuracy were observed for 3 bp modifications in the “CDS-like” region and shuffling of the “3′UTR-like” region.

This stability is highly significant. It demonstrates that TRANSAID’s classification of a transcript as non-coding is not based on the mere absence of start/stop codon patterns, but on a more holistic assessment of whether the sequence possesses the genuine, complex features compatible with translation. The fact that random mutations do not easily convert a non-coding transcript into a coding one in the model’s view reflects biological reality and highlights the sophistication of the features learned by TRANSAID. Collectively, these perturbation experiments confirm that TRANSAID has captured several fundamental principles of translation machinery, including the triplet codon architecture, the regulatory importance of the 5′UTR, and the contextual features that robustly distinguish translatable from non-translatable RNA molecules.

### 3.3 TRANSAID demonstrates robust cross-species generalization

**FIGURE 4:**
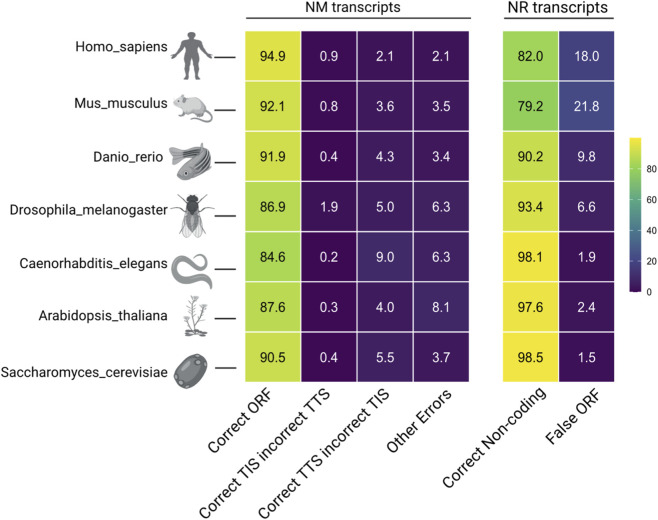
Comprehensive cross-species performance of TRANSAID. A heatmap summarizing the performance of the human-trained TRANSAID model across six additional eukaryotic species, ordered by decreasing evolutionary relatedness to humans. Performance is stratified into protein-coding (NM, left panel) and non-coding (NR, right panel) transcripts. For NM transcripts, the “Correct ORF” metric demonstrates robust accuracy across vertebrates with a gradual decline in distant species. Conversely, for NR transcripts, the “Correct Non-coding” accuracy improves significantly as species diverge from humans. Numerical values represent the percentage of transcripts in each category, with color intensity corresponding to the percentage value.

A critical measure of a predictive model’s utility is its ability to generalize beyond the data it was trained on. To assess the extent to which our human-trained TRANSAID model learned evolutionarily conserved principles of translation, we evaluated its performance on the transcriptomes of six additional eukaryotic species, spanning a wide evolutionary divergence from humans: *Mus musculus*, *Danio rerio*, *Drosophila melanogaster*, *Caenorhabditis elegans*, *Arabidopsis thaliana*, and *Saccharomyces cerevisiae*. The comprehensive results of this cross-species analysis are summarized in Figure 4.

For the prediction of protein-coding (NM) transcripts, TRANSAID demonstrated remarkable and robust performance, underscoring the deep conservation of the core translation machinery across eukaryotes. The model achieved a “Correct ORF” (defined as a prediction where the final ORF, after adjustment by the integrated scoring system, correctly matches the annotated TIS and TTS) prediction rate of 92.1% in *Mus musculus*, a performance nearly on par with that observed in humans (94.9%). As expected, this accuracy exhibited a gradual and modest decline with increasing evolutionary distance, remaining high in *Danio rerio* (91.9%) and *Drosophila melanogaster* (86.9%), and still maintaining strong performance in the highly divergent *Caenorhabditis elegans* (84.6%), *Arabidopsis thaliana* (87.6%), and *Saccharomyces cerevisiae* (90.5%) species. This trend reflects the subtle divergence in species-specific translation regulatory mechanisms, such as codon usage bias and local sequence motifs, yet affirms that the fundamental features learned by TRANSAID are largely universal across the eukaryotic domain.

Intriguingly, the model’s performance on non-coding (NR) transcripts revealed an inverse trend. The accuracy of “Correct Non-coding” prediction, a measure of the model’s ability to correctly reject non-translatable sequences, improved significantly with increasing evolutionary distance from humans (Figure 4, right panel). While the accuracy for the closely related *Mus musculus* was 79.2%, it rose to 90.2% in *Danio rerio*, 93.4% in *Drosophila melanogaster*, and peaked at over 97% in the most distant species, *Caenorhabditis elegans* (98.1%), *Arabidopsis thaliana* (97.6%), and *Saccharomyces cerevisiae* (98.5%).

This seemingly counterintuitive pattern provides a key insight into the model’s learning process. The human-trained model has learned to distinguish human coding sequences from human non-coding sequences. As species diverge, their non-coding RNAs tend to evolve much more rapidly in sequence and structure than their protein-coding genes (Ulitsky, 2016). Consequently, the non-coding transcripts of distant species like *Saccharomyces cerevisiae* become increasingly dissimilar to the complex, signal-rich patterns of human protein-coding transcripts. For the model, these highly divergent NR sequences present a less ambiguous negative signal, making them easier to classify correctly as non-coding. Conversely, the NR transcripts of closer relatives like *Mus musculus* may retain more sequence artifacts or conserved non-coding elements that superficially resemble features of human coding regions, thus posing a more difficult classification challenge. This robust cross-species performance not only highlights TRANSAID’s powerful generalization capabilities but also underscores its sophisticated capture of evolutionarily conserved features of the translation machinery.

### 3.4 TRANSAID outperforms state-of-the-art tools in key aspects

To rigorously assess TRANSAID’s performance in the context of existing technologies, we conducted a comprehensive benchmark against three state-of-the-art tools: TranslationAI, a contemporary deep learning framework; GeneMarkS-T, a widely-used statistical method based on Hidden semi-Markov Models; and TITER, a specialized deep learning tool for TIS prediction. We first performed a qualitative comparison of their functional capabilities, followed by a quantitative performance evaluation on the same independent test set across all seven eukaryotic species.

**FIGURE 5:**
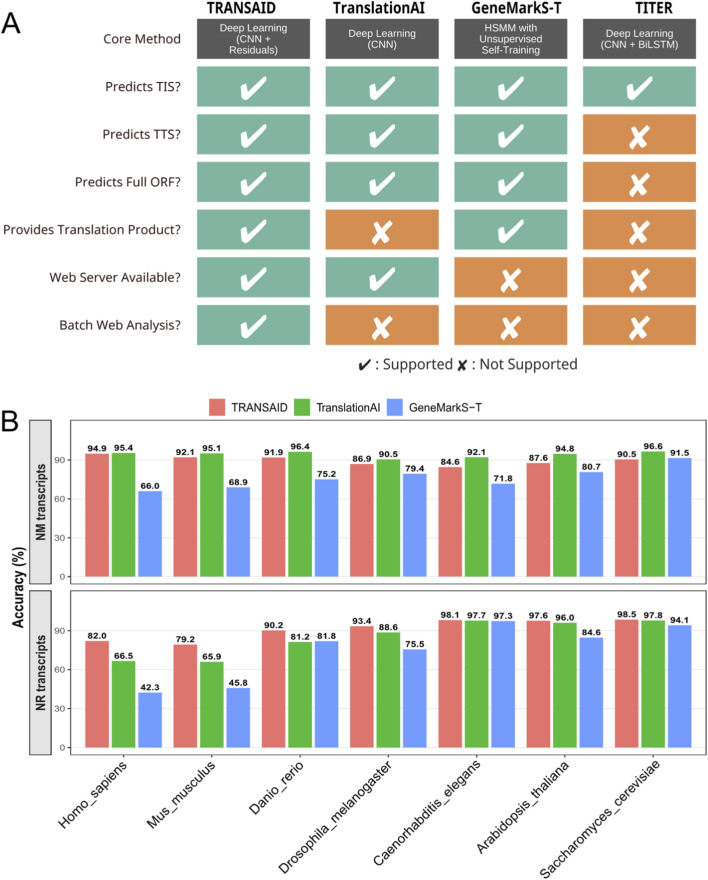
Comparative Analysis of ORF Prediction Tools. **(A)** A feature comparison of TRANSAID against leading ORF prediction tools. Capabilities are evaluated across methodology and functional outputs. Checkmarks (✔) indicate full support, while crosses (✘) indicate a lack of support. **(B)** Quantitative performance comparison of TRANSAID, TranslationAI, and GeneMarkS-T across seven eukaryotic species. The top panel measures “Correct ORF” prediction accuracy on NM transcripts, where both deep learning models (TRANSAID and TranslationAI) significantly outperform the statistical model-based GeneMarkS-T. The bottom panel measures “Correct Non-coding” accuracy on NR transcripts, where TRANSAID demonstrates consistently superior performance, particularly in vertebrates.

The functional comparison, summarized in Figure 5A, highlights significant differences in the scope and utility of each tool. While all methods are capable of predicting TIS, their end-to-end capabilities vary substantially. Specialized tools like TITER focus exclusively on TIS identification and do not provide predictions for TTS or the full ORF. In contrast, TRANSAID, TranslationAI, and GeneMarkS-T are all designed to predict complete ORFs. However, among these, only TRANSAID and GeneMarkS-T are designed for local execution on large datasets, as TranslationAI’s web server limits batch processing. Critically, TRANSAID is the only tool in this comparison that provides a comprehensive, end-to-end workflow, integrating a user-friendly web server for both single and batch analysis with the direct output of translated protein products, a feature essential for downstream proteomic analyses.

Quantitative benchmarking on the independent test set further revealed TRANSAID’s superior performance in key areas (Figure 5B). For the task of predicting ORFs in protein-coding (NM) transcripts, we measured the percentage of “Perfect ORF” predictions, where both the TIS and TTS must be identified with single-nucleotide precision. The results, shown in the top panel of Figure 5B, demonstrate a clear performance advantage for deep learning-based methods. Both TRANSAID and TranslationAI consistently and significantly outperformed the statistical model-based GeneMarkS-T across all seven species. For instance, in *Homo sapiens*, TRANSAID achieved a “Perfect ORF” rate of 94.9%, comparable to TranslationAI’s 95.4%, while both were substantially higher than GeneMarkS-T’s 66.0%. This trend holds across the evolutionary spectrum, underscoring the power of deep learning architectures to capture the complex sequence patterns governing translation boundaries more effectively than traditional probabilistic models.

The most striking performance difference was observed in the critical task of correctly identifying non-coding (NR) transcripts, a known challenge for translation prediction tools. As shown in the bottom panel of Figure 5B, TRANSAID demonstrated consistently superior accuracy in this domain. In *Homo sapiens*, TRANSAID correctly classified 82.0% of NR transcripts as non-coding, outperforming both TranslationAI (66.5%) and GeneMarkS-T (42.3%). This advantage was also in the closely related *Mus musculus*, where TRANSAID’s accuracy (79.2%) was significantly higher than that of TranslationAI (65.9%) and GeneMarkS-T (45.8%). While the performance gap narrowed in more evolutionarily distant species where NR sequences are more distinct, TRANSAID maintained a competitive or leading edge across the board. This superior specificity in distinguishing non-coding transcripts is a direct result of TRANSAID’s mixed-training strategy and robust feature learning, positioning it as a more reliable tool for transcriptome-wide annotation, particularly in complex vertebrate genomes where the potential for false positive ORF predictions is high.

### 3.5 Experimental validation and discovery of novel coding events

Beyond computational benchmarks, a crucial test of a prediction tool’s real-world utility is its ability to identify translated products that can be validated by experimental evidence. To assess TRANSAID’s performance in this capacity, we applied it to two distinct discovery-oriented tasks: identifying proteins from novel, long-read transcript isoforms and exploring the cryptic coding potential of annotated non-coding (NR) transcripts.

**FIGURE 6:**
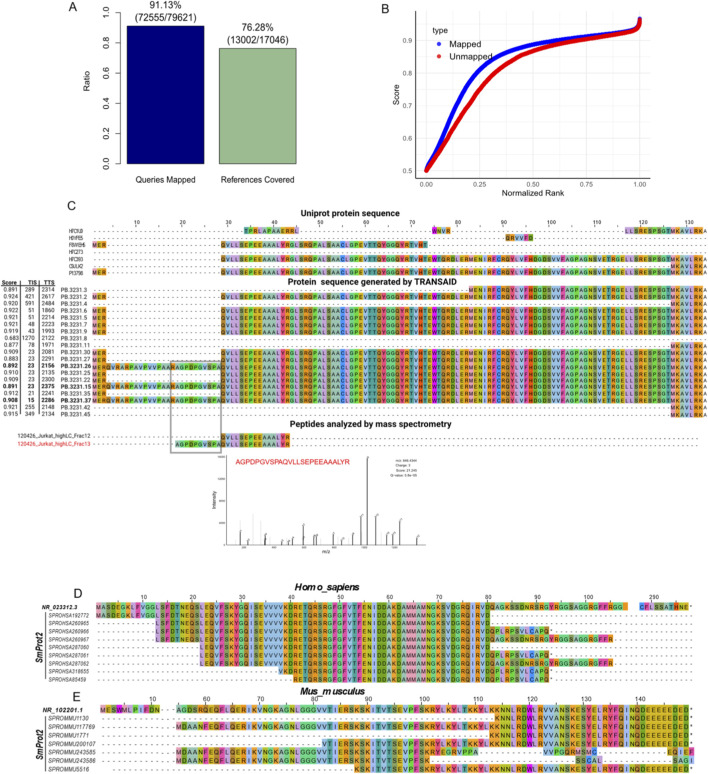
Validation of TRANSAID Predictions with Proteomic Evidence. **(A)** Coverage analysis of TRANSAID’s predictions on novel Jurkat T-cell transcripts against mass spectrometry (MS) data. 76.28% of the non-redundant proteins predicted by TRANSAID were supported by peptide evidence (“References Covered”), and 91.13% of all experimentally identified peptides mapped to our predicted protein set (“Queries Mapped”). **(B)** Distribution of Integrated_Score values for MS-validated (“Mapped”) versus unvalidated (“Unmapped”) proteins, demonstrating that our scoring system effectively prioritizes true positives. **(C)** A case study of novel isoform discovery for the APEH gene. TRANSAID correctly predicted a protein isoform containing a unique sequence from a retained intron (middle panel), which was subsequently confirmed by a unique peptide identified in the MS data (bottom panel). **(D,E)** Validation of cryptic sORF predictions from non-coding (NR) transcripts via genomic coordinate analysis. Representative multiple sequence alignments show that protein sequences translated from TRANSAID-predicted ORFs in human **(D)** and mouse **(E)** NR transcripts exhibit high identity to experimentally validated small proteins from the SmProt database that are annotated at the same genomic loci.

First, we evaluated TRANSAID’s ability to annotate the proteome from a complex, experimentally derived transcriptome. We used a publicly available dataset from Jurkat T-cells comprising both PacBio Iso-Seq full-length transcripts and corresponding high-resolution mass spectrometry (MS) data (Miller et al., 2022). After processing the novel transcripts with TRANSAID and performing redundancy removal, we generated a custom database of 17,046 predicted protein sequences. We then mapped the experimentally identified MS peptides from the original study against this database. The results showed a strong validation rate: a significant 76.28% (13,002 out of 17,046) of our predicted proteins were supported by direct peptide evidence. Conversely, 91.13% of all experimentally identified peptides mapped back to our predicted protein set, indicating high coverage (Figure 6A). Furthermore, the integrated scores of the MS-validated proteins were significantly higher than those of the unvalidated proteins, suggesting our scoring system effectively prioritizes true positives (Figure 6B).

This validation extended to the discovery of novel protein isoforms. For example, TRANSAID successfully predicted protein sequences for three novel isoforms of the APEH gene that were not present in the UniProt database. These isoforms contained a unique amino acid sequence derived from a retained intron. Critically, we identified a peptide in the MS data that mapped uniquely to this novel intron-derived region, providing direct experimental confirmation of a previously unannotated translation event (Figure 6C). This demonstrates TRANSAID’s power in augmenting proteomic databases by accurately translating novel isoforms discovered through long-read sequencing.

Second, we investigated the model’s potential to uncover novel coding events from transcripts conventionally considered non-coding. To move beyond sequence similarity and provide a more stringent validation, we implemented a genomic coordinate-based analysis. We intersected the genomic coordinates of high-confidence ORFs predicted by TRANSAID within NR transcripts with those of experimentally validated small proteins from the SmProt2 High-Confidence database.

The results, summarized in the revised Supplementary Table S1, confirm that a subset of these predictions corresponds to *bona fide*, annotated sORFs. In *Homo sapiens*, we identified 114 predicted NR-ORFs that directly overlap with known sORF entries, corresponding to a 7.42% validation rate against the comprehensive SmProt human dataset. In *Mus musculus*, 8 such events were confirmed (0.60% validation rate). The lower validation rate in mouse and the absence of matches in species like *Drosophila melanogaster* are likely attributable to the significantly smaller number of curated sORFs available in SmProt for these organisms (383 for mouse and only 11 for fruit fly, compared to 8,654 for human), rather than a lack of model performance.

To visually confirm the validity of these coordinate-based matches, we performed multiple sequence alignments of the translated protein sequences. As shown in representative examples for both *Homo sapiens* (Figure 6D) and *Mus musculus* (Figure 6E), the amino acid sequences predicted by TRANSAID show perfect or near-perfect identity to the curated SmProt entries, providing unequivocal evidence of correct predictions. The complete list of all 122 validated NR transcripts is available in Supplementary File 2 (SmProt2_matched_NR.transcripts.xlsx).

This rigorous, location-aware analysis reframes a significant portion of the model’s apparent “false positives” as valuable and validated discoveries. It highlights TRANSAID’s capability as a powerful tool for exploring the cryptic coding landscape of the non-coding genome, moving beyond annotation to genuine discovery.

## 4 Discussion

In this study, we introduced TRANSAID, a novel deep learning framework that addresses several persistent challenges in computational translation prediction. Our comprehensive evaluations demonstrate that TRANSAID achieves exceptional performance in identifying translation sites across diverse transcript types and species. Through a combination of a robust model architecture, a strategic mixed-training approach, and a sophisticated integrated scoring system, whose parameters were systematically optimized in a data-driven manner, TRANSAID offers significant advantages over existing methods.

A key innovation of TRANSAID is its ability to process full-length transcripts in an end-to-end manner, simultaneously predicting both TIS and TTS pairs while enforcing biological constraints. This holistic approach contrasts with specialized tools like TITER, which focus solely on TIS identification, and circumvents the limitations of window-based methods that may fail to capture long-range dependencies. Furthermore, the strategic inclusion of both protein-coding (NM) and non-coding (NR) transcripts during training proved to be a critical decision. As our results show (Figure 5B), this mixed-training strategy substantially improves the model’s ability to distinguish genuine translation events from spurious sequence patterns, leading to a marked reduction in false positive predictions on NR transcripts compared to both TranslationAI and the statistical model-based GeneMarkS-T. This enhanced specificity is crucial for accurate transcriptome-wide annotation, particularly in complex genomes with vast non-coding regions.

Our sequence perturbation experiments provided compelling insights into the model’s inner workings, revealing that TRANSAID has learned fundamental principles of translation beyond superficial pattern matching (Figure 3). The model’s extreme sensitivity to frameshift mutations, contrasted with its tolerance for in-frame modifications, demonstrates its implicit understanding of the triplet genetic code. Similarly, the differential impact of 5′UTR versus 3′UTR modifications aligns with the established biological understanding that 5′UTR regions contain critical regulatory elements for translation initiation (Hinnebusch et al., 2016). These findings suggest that TRANSAID has developed a sophisticated, context-aware representation of translation-compatible features. This learned knowledge base likely contributes to its strong cross-species generalization. Despite being trained primarily on human data, the model maintained robust performance across organisms from mammals to fungi (Figure 4), This indicates that TRANSAID has captured deeply conserved, fundamental features of the translation machinery that are shared across a vast range of eukaryotic life (Merrick and Pavitt, 2018).

Perhaps one of the most significant applications of TRANSAID is its potential as a discovery engine for novel coding events. The synergy between long-read sequencing and accurate *de novo* translation prediction opens new frontiers for proteogenomics. Our analysis of experimental data from Jurkat T-cells demonstrated this capability, where TRANSAID not only validated a high percentage (76.28%) of its predictions with mass spectrometry evidence but also successfully identified previously unannotated protein isoforms arising from events like intron retention (Figure 6). Moreover, our investigation into the ORFs predicted within NR transcripts provides intriguing, albeit preliminary, evidence for the discovery of cryptic sORFs. The finding that a substantial fraction (7.42% in humans) of these predicted micropeptides show homology to proteins in SmProt (Supplementary Table S1) suggests that many of the model’s apparent “false positives” may in fact be biologically significant, unannotated coding events. While further experimental validation is required, this highlights TRANSAID’s potential to systematically mine the non-coding transcriptome for novel functional elements, a task of growing importance in functional genomics (Ruiz-Orera and Albà, 2019).

Despite its strong performance, TRANSAID has several limitations that represent avenues for future development. First, the current model is primarily trained to recognize canonical AUG start codons. While it can identify some alternative initiation events, its sensitivity could be enhanced through explicit training on experimentally verified non-AUG TIS and re-initiation sites, such as those cataloged from ribosome profiling studies (Kahles et al., 2018). Second, our framework does not yet explicitly model complex translation phenomena like programmed ribosomal frameshifting or stop-codon read-through, which contribute to proteome diversity. Incorporating models of these events would be a valuable future enhancement. Third, our data-driven optimization revealed that some canonical biological features like GC content become redundant when paired with a powerful deep learning model. This suggests that future work could focus on incorporating more complex, orthogonal information, such as predicted RNA secondary structures (Lin et al., 2022),which may provide novel predictive power. Finally, its application to prokaryotic systems would require modifications to account for distinct mechanisms like Shine-Dalgarno sequence-based initiation.

In conclusion, TRANSAID represents a significant advance in computational translation prediction. By addressing the critical limitations of existing approaches—including training data bias, the inability to process full-length transcripts, and a lack of integrated biological constraints—TRANSAID provides a powerful, accurate, and versatile tool for the scientific community. Its demonstrated high accuracy, robust cross-species applicability, and potential for discovering novel coding events from both alternative isoforms and the non-coding genome underscore its value in advancing our understanding of translation regulation and discovering novel protein products in diverse biological contexts.

## Data Availability

The original contributions presented in the study are included in the article/Supplementary Material, further inquiries can be directed to the corresponding author.
