# Supplementary material for: TRANSAID: a hybrid deep learning framework for translation site prediction with integrated biological feature scoring
Source: Front Bioinform. 2026 Jan 19;5:1676149. doi: 10.3389/fbinf.2025.1676149 (PMC12862215; doi:10.3389/fbinf.2025.1676149)
Supplement: Supplementary file 1 [file DataSheet1.pdf]

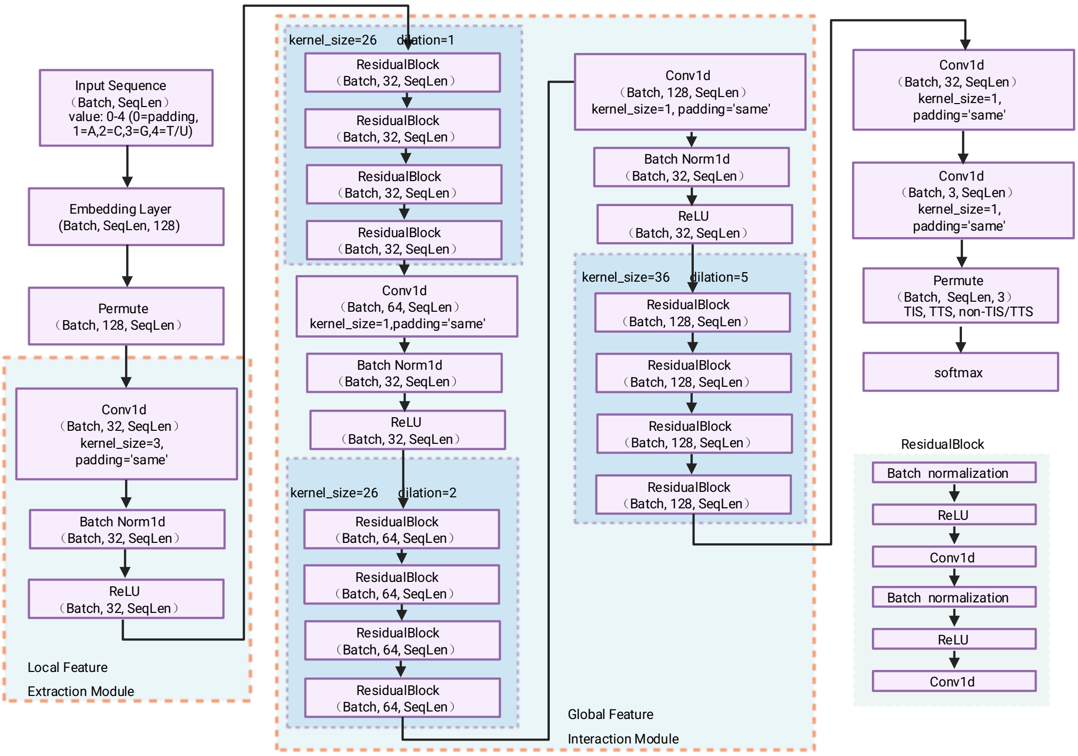

Supplementary Figure 1: TRANSAID Model Architecture.

The model processes integer-encoded nucleotide sequences (0-4) through four main parts. **1) Local Feature Extraction Module:** Initiates by embedding the input sequence into a 128-dimensional continuous vector space. After a permutation, a 1D convolutional layer (kernel_size=3) extracts fundamental local sequence patterns, followed by Batch Normalization and ReLU activation, reducing the feature map to 32 channels; **2) Global Feature Interaction Module:** Comprises three sequential stages of four ResidualBlock_v2 units each, interspersed with 1x1 convolutions for channel dimension expansion. Stage 1 (32 channels) uses kernel_size=26, dilation=1. It is followed by a 1x1 convolution (with BN and ReLU) that expands features to 64 channels. Stage 2 (64 channels) employs kernel_size=26, dilation=2. Another 1x1 convolution (with BN and ReLU) then expands features to 128 channels. Stage 3 (128 channels) utilizes kernel_size=36, dilation=5. These stages progressively capture long-range dependencies through increasing dilation rates; **3) Output Decoding Module:** Two final 1x1 convolutional layers reduce the 128-dimensional high-level features sequentially to 32, then to 3 channels, representing the logits for TIS, TTS, and non-TIS/TTS classes for each nucleotide position. The output is then permuted.

4)ResidualBlock: Each block implements a pre-activation residual structure with Batch Normalization, ReLU, and 1D convolutions, ensuring stable training and effective gradient flow.

A

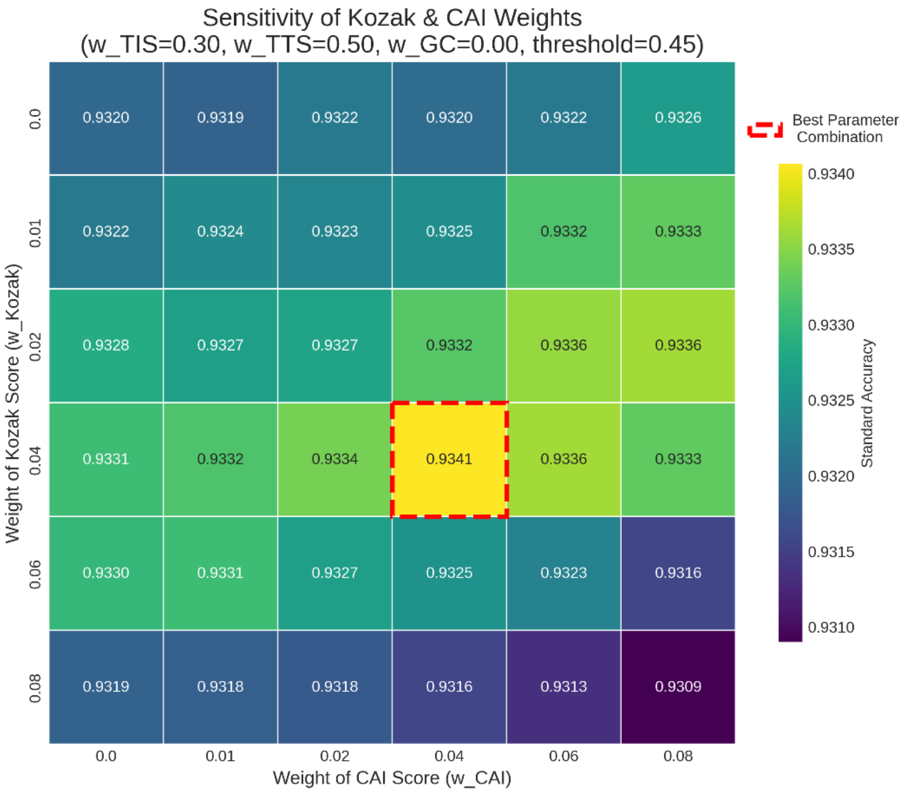
Sensitivity of Kozak & CAI Weights
(w_TIS=0.30, w_TTS=0.50, w_GC=0.00, threshold=0.45)
Weight of Kozak Score (w_Kozak)
Weight of CAI Score (w_CAI)
0.0 0.01 0.02 0.04 0.06 0.08
0.0 0.01 0.02 0.04 0.06 0.08
0.9320 0.9319 0.9322 0.9320 0.9322 0.9326
0.9322 0.9324 0.9323 0.9325 0.9332 0.9333
0.9328 0.9327 0.9327 0.9332 0.9336 0.9336
0.9331 0.9332 0.9334 0.9341 0.9336 0.9333
0.9330 0.9331 0.9327 0.9325 0.9323 0.9316
0.9319 0.9318 0.9318 0.9316 0.9313 0.9309
Best Parameter Combination
Standard Accuracy
0.9340
0.9335
0.9330
0.9325
0.9320
0.9315
0.9310

B

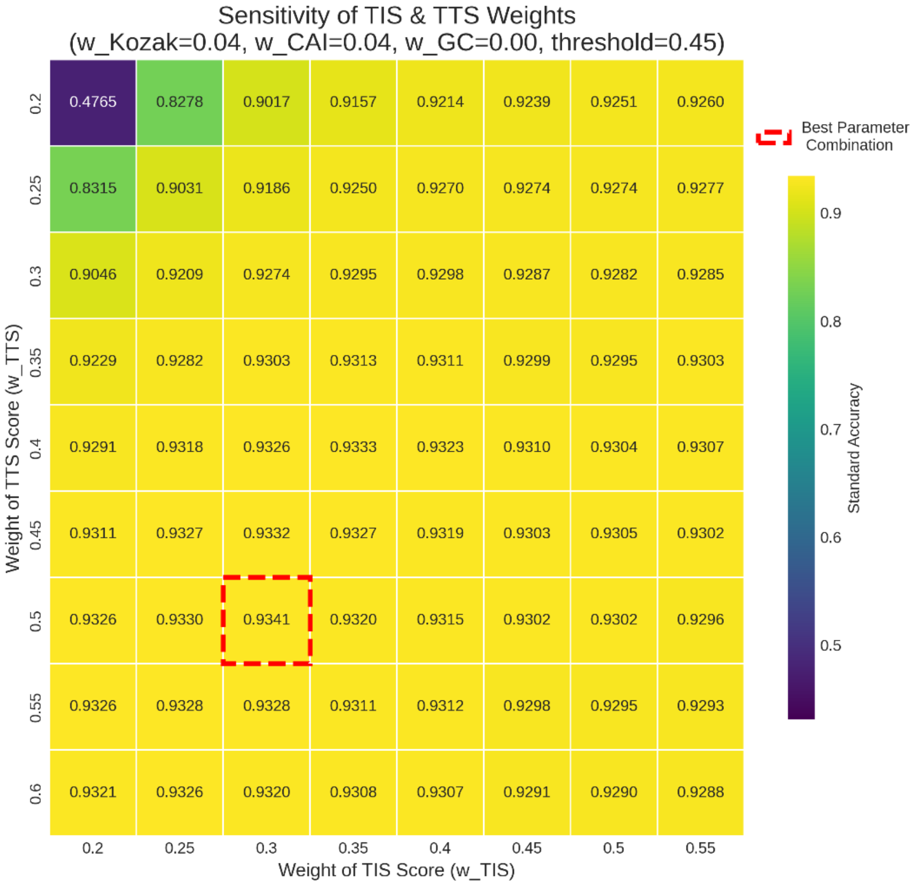
Sensitivity of TIS & TTS Weights
(w_Kozak=0.04, w_CAI=0.04, w_GC=0.00, threshold=0.45)
Weight of TTS Score (w_TTS)
Weight of TIS Score (w_TIS)
0.2 0.25 0.3 0.35 0.4 0.45 0.5 0.55 0.6
0.2 0.25 0.3 0.35 0.4 0.45 0.5 0.55
0.4765 0.8278 0.9017 0.9157 0.9214 0.9239 0.9251 0.9260
0.8315 0.9031 0.9186 0.9250 0.9270 0.9274 0.9274 0.9277
0.9046 0.9209 0.9274 0.9295 0.9298 0.9287 0.9282 0.9285
0.9229 0.9282 0.9303 0.9313 0.9311 0.9299 0.9295 0.9303
0.9291 0.9318 0.9326 0.9333 0.9323 0.9310 0.9304 0.9307
0.9311 0.9327 0.9332 0.9327 0.9319 0.9303 0.9305 0.9302
0.9326 0.9330 0.9341 0.9320 0.9315 0.9302 0.9302 0.9296
0.9326 0.9328 0.9328 0.9311 0.9312 0.9298 0.9295 0.9293
0.9321 0.9326 0.9320 0.9308 0.9307 0.9291 0.9290 0.9288
Best Parameter Combination
Standard Accuracy
0.9
0.8
0.7
0.6
0.5

C

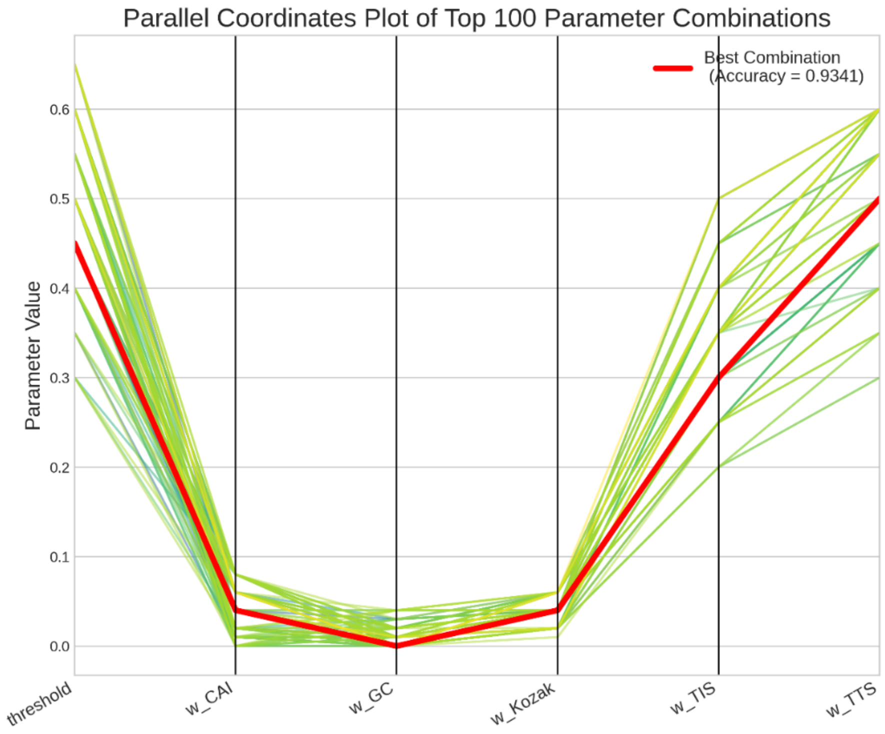

Supplementary Figure 2: Data-Driven Optimization and Sensitivity Analysis of the Integrated Scoring System Parameters.

The optimal weights and threshold for the Integrated_Score were determined by a comprehensive grid search on the independent validation set, maximizing for Standard Accuracy. **(A) Sensitivity analysis of biological feature weights.** This heatmap displays the Standard Accuracy as a function of w_Kozak and w_CAI, while w_TIS, w_TTS, w_GC, and threshold are fixed at their optimal values. The analysis shows that incorporating these features at low weights provides a measurable performance improvement. The optimal combination is highlighted by a dashed red box. **(B) Sensitivity analysis of TIS and TTS weights.** This heatmap visualizes the impact of the dominant w_TIS and w_TTS parameters, with other parameters fixed at their optimal values. The performance peak at w_TIS=0.30 and w_TTS=0.50 (highlighted) informed the final parameter selection. **(C) Parallel coordinates plot of the top 100 parameter combinations.** Each line represents a parameter set, colored by its accuracy. The plot provides a holistic view of the high-performance parameter space, with the single best-performing combination highlighted in red.

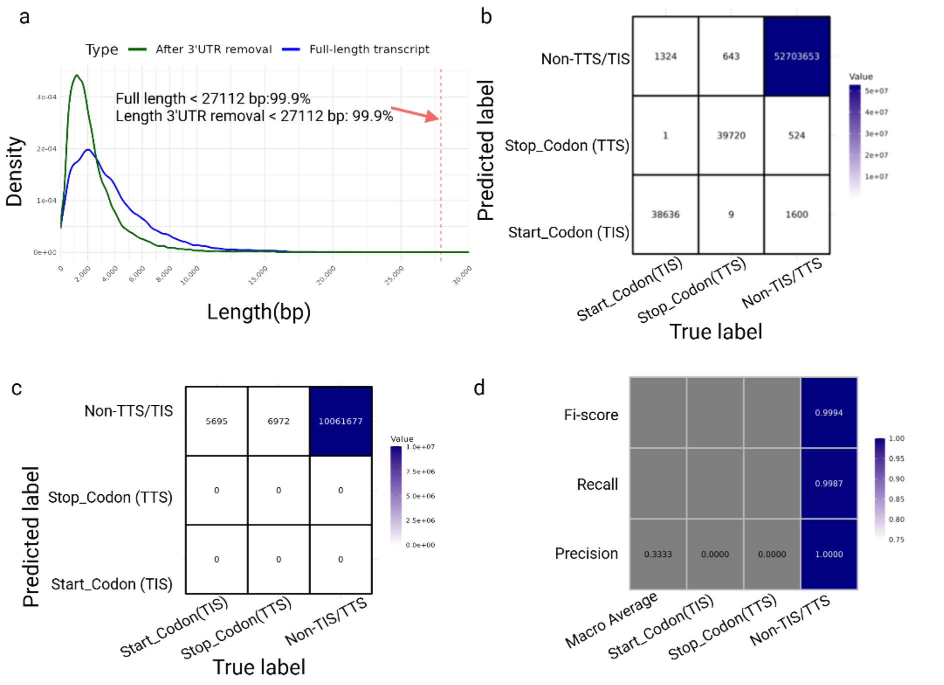

Supplementary Figure 3: Model Training Data and Initial Performance. (A) Length distribution of human transcripts. The maximum input length for TRANSAID was set to 27,112 nt to cover 99.9% of all transcripts. (B) Confusion matrix for the TrainNMonly model evaluated on the NM test set, showing precise TIS/TTS identification at the nucleotide level. (C, D) Performance of the TrainNMonly model on NR test transcripts, revealing an elevated false positive rate and highlighting the necessity of including NR transcripts in the training data.

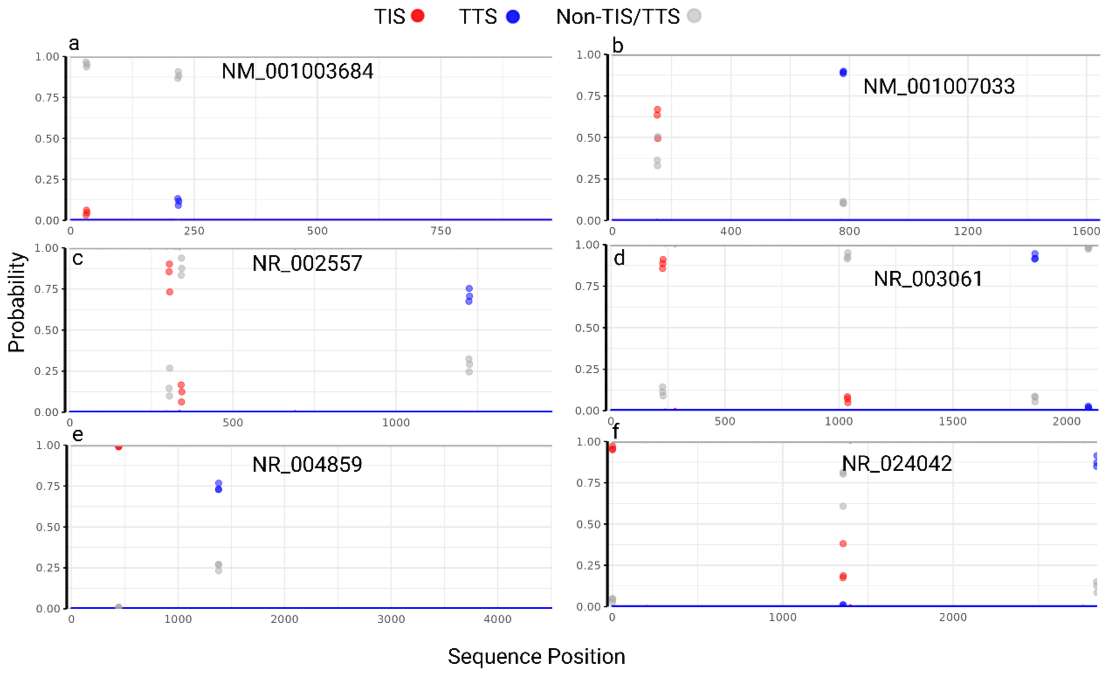

Supplementary Figure 4: Analysis of Misclassified Transcripts. (a, b) Probability distribution plots for protein-coding transcripts (NM_001003684, NM_001007033) incorrectly classified as non-coding. Despite the misclassification, elevated probabilities are evident at the true TIS/TTS positions. (c-f) Probability distributions for non-coding transcripts incorrectly predicted to contain ORFs. These plots often reveal sequence patterns that mimic translation signals but lack proper biological context, demonstrating the potential for filtering based on features like codon structure.

Supplementary Table 1: Validation of Predicted ORFs in Non-Coding (NR) Transcripts via Genomic Coordinate Overlap with the SmProt Database. To stringently validate the biological significance of ORFs predicted within NR transcripts, we performed a genomic coordinate-based analysis instead of a sequence homology search. The genomic coordinates of ORFs confidently predicted by TRANSAID were intersected with the coordinates of experimentally validated small proteins from the SmProt2 High-Confidence Set. A prediction was considered validated only if a direct and substantial overlap was found. This rigorous analysis confirms that a subset of TRANSAID's predictions on NR transcripts correspond to bona fide sORFs. The lower validation rate in species like *Mus musculus* compared to *Homo sapiens* is likely attributable to the significantly smaller number of curated sORFs available in the SmProt database for those species (383 entries for mouse vs. 8,654 for human).

Species	*Total Predicted NR*	*Total SmProt Seq*	*Supported by SmProt*	*Supported Rate (%)*
Homo_sapiens	1535	8654	114	7.42
Mus_musculus	1323	383	8	0.60
Drosophila_melanogaster	78	11	0	0
